# Supplementary material for: Economic burden of typhoid among hospitalised children in Kenya
Source: BMJ Glob Health. 2026 Aug 11;11(8):e023131. doi: 10.1136/bmjgh-2025-023131 (PMC13475079; doi:10.1136/bmjgh-2025-023131)
Supplement: Supplementary file 2 [file bmjgh-11-8-s002.docx]

### BMJ Global Health Author Reflexivity Statement

Adapted from Morton, B., Vercueil, A., Masekela, R., Heinz, E., Reimer, L., Saleh, S., Kalinga, C., Seekles, M., Biccard, B., Chakaya, J., Abimbola, S., Obasi, A. and Oriyo, N. (2022), Consensus statement on measures to promote equitable authorship in the publication of research from international partnerships. Anaesthesia, 77: 264-276. <https://doi.org/10.1111/anae.15597>

| **Study conceptualisation** | |
| --- | --- |
| 1. How does this study address local research and policy priorities? | The study was developed to fill a critical gap in Kenyan data on the cost of treating typhoid, which was needed for the economic evaluation of the typhoid conjugate vaccine, a government priority. |
| 2. How were local researchers involved in study design? | Local researchers were involved in the conceptualization (EB, SO, PM), study design (PM, SO, IW, FO, and EB), framing the research question, data collection, and data analysis (PM, SO, IW, FO, and EB). |
| **Research management** | |
| 3. How has funding been used to support the local research team(s)? | Funding for this work supported PM and SO’ s salaries, both based at KEMRI-Wellcome Trust Research Programme |
| **Data acquisition and analysis** | |
| 4. How are research staff who conducted data collection acknowledged? | They are co-authors in the study. |
| 5. How have members of the research partnership been provided with access to study data? | All members of the partnership have access to the data. |
| 6. How were data used to develop analytical skills within the partnership? | Research members worked collaboratively facilitating the sharing of skills across multidisciplinary team members. |
| **Data interpretation** | |
| 7. How have research partners collaborated in interpreting study data? | Research partners scheduled meetings to deliberate and agree on analytical approaches, provide support where needed. |
| **Drafting and revising for intellectual content** | |
| 8. How were research partners supported to develop writing skills? | They research team comprised of several senior scientists with established writing skills. These scientists worked with early career staff to support the writing. |
| 9. How will research products be shared to address local needs? | Findings from this analysis will be disseminated locally and internationally through presentations with local stakeholders, conferences, and publication. |
| **Authorship** | |
| 10. How is the leadership, contribution and ownership of this work by LMIC researchers recognised within the authorship? | All co-authors except for LC are LMIC researchers. |
| 11. How have early career researchers across the partnership been included within the authorship team? | The lead author (PM) is an early career researcher. |
| 12. How has gender balance been addressed within the authorship? | One author is female (SO) and the remaining are male. |
| **Training** | |
| 13. How has the project contributed to training of LMIC researchers? | Funding for this work is planned to support PM’s PhD studentship. PM’s studentship will be supervised by SO, LC and EB |
| **Infrastructure** | |
| 14. How has the project contributed to improvements in local infrastructure? | The project has not directly contributed to improvements in local infrastructure. |
| **Governance** | |
| 15. What safeguarding procedures were used to protect local study participants and researchers? | Through ethical approval and obtaining informed consent. |
